# Supplementary material for: Causal Relationship between Aging and Anorexia Nervosa: A White-Matter-Microstructure-Mediated Mendelian Randomization Analysis
Source: Biomedicines. 2024 Aug 16;12(8):1874. doi: 10.3390/biomedicines12081874 (PMC11351342; doi:10.3390/biomedicines12081874)
Supplement: Supplementary file 1 [file biomedicines-12-01874-s001.zip › Supplementary Figure/Figure S8/Leaveoneout plot of MR analysis for TL to ubm-b-1579.pdf]

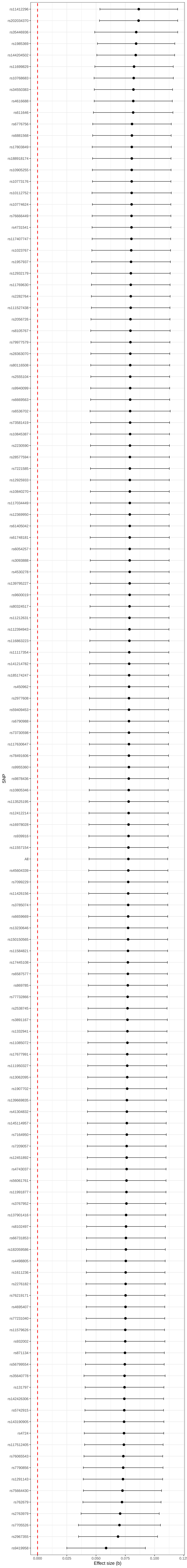

rs11412296
rs202034370
rs35446936
rs1985369
rs144204502
rs11699829
rs10768663
rs34050383
rs4616688
rs611646
rs6776756
rs6881568
rs17803849
rs188918174
rs10905255
rs10773176
rs10112752
rs10774624
rs76666449
rs4731541
rs117407747
rs1023767
rs1957937
rs12932179
rs11769630
rs2282764
rs111527438
rs2056726
rs8105767
rs79977579
rs28363070
rs80116508
rs2555104
rs9940099
rs6669563
rs6536702
rs73581419
rs10845387
rs2230590
rs28577594
rs7221585
rs12925933
rs10840270
rs117034449
rs12369950
rs61405042
rs61748181
rs6054257
rs3093888
rs4530278
rs139795227
rs9600019
rs80324517
rs11212631
rs112394943
rs116863223
rs11117354
rs141214782
rs185174247
rs450962
rs2977608
rs59409453
rs6790988
rs73730598
rs117630647
rs78491606
rs9955360
rs9878436
rs10805346
rs113525195
rs12412214
rs16978028
rs939916
rs11557154
A8
rs45604339
rs7099229
rs11426156
rs3785074
rs6659669
rs13230646
rs150150565
rs11584821
rs17445108
rs6587577
rs869785
rs77732866
rs2538745
rs3891167
rs1332941
rs11085072
rs17677991
rs111950327
rs13062095
rs1907702
rs139669835
rs41304832
rs145114957
rs7164950
rs7209057
rs12451892
rs4743037
rs56061761
rs11991877
rs3767952
rs137901416
rs8102497
rs66731853
rs182059586
rs4498805
rs1611236
rs2276182
rs76219171
rs4695407
rs77231040
rs11579626
rs932002
rs871134
rs56799554
rs35640778
rs131797
rs142426306
rs5742915
rs143190905
rs4724
rs117512405
rs76065543
rs7780656
rs12911143
rs75664430
rs762679
rs2763979
rs7705526
rs2967355
rs9419958
SNP
0.000
0.025
0.050
0.075
0.100
0.125
Effect size (b)
